# Pot Song as a Novel Cancer Communication Intervention: Lessons Learned from Developing, Implementing, and Evaluating a Culturally Grounded Intervention for Breast Cancer Education in Rural Bangladesh

**DOI:** 10.1007/s13187-021-02111-1

**Published:** 2021-11-30

**Authors:** Aantaki Raisa, Anthony J. Roberto, Richard R. Love, Heather L. Story Steiness, Reza Salim, Janice L. Krieger

**Affiliations:** 1 University of Florida, Gainesville, FL USA; 2 Arizona State University, Tempe, AZ USA; 3 Marquette University, Milwaukee, WI USA; 4 University of Phoenix, Tempe, AZ USA; 5 Amader Gram, Dhaka, Bangladesh

**Keywords:** Cancer education, Health communication, Intervention, Breast problems, Bangladesh, Cultural grounding, Rural health, Pot song

## Abstract

Targeted public education may offer an approach to achieving more effective treatment in countries like Bangladesh, where breast cancer is a leading cause of cancer death in women. Effective cancer education interventions address the target population’s cultural and contextual needs. However, there is little published literature to guide the development of educational cancer interventions in a region where lack of resources combined with cultural stigma about cancer contribute to poor breast health outcomes for women. The goal of the current study was to design, test, and evaluate a culturally grounded intervention to promote breast problem care among women in rural Bangladesh. The current manuscript first describes the process of formative evaluation that led to the development of the intervention, including decisions about the audience, message construction, and mode of intervention delivery. Second, we describe the testing process, including process and outcome evaluation. Finally, we describe the lessons learned from the process. We conclude with recommendations related to cultural grounding for message development, questionnaire design, data collection procedures, and analysis.

## Introduction

Theory-driven, culturally grounded cancer education programs can be vital in increasing breast cancer (BCa) awareness and improving breast care, especially among underserved populations in low-resource countries where BCa mortality-incidence ratios are double that reported in high-income countries [1, 2]. Cultural grounding is a process that ensures cancer education interventions reflect the culture of the intended audience. This is accomplished through the active participation of members of the intended audience in the development and dissemination process [3]. The process of cultural grounding helps to create educational activities and materials that are patient-centric, meaning that they have been linguistically and culturally translated to be appropriate for patients [4]. Cultural grounding is particularly important in cancer education, as cancer is conceptualized differently across cultures with specific stigmas, myths, and taboos [5].

The process for culturally grounding cancer educational interventions has largely been documented in high-resource countries like the USA; thus, little is known about facilitators of and barriers to the process in non-Western, low-resource countries [6]. The country site for the current study is Bangladesh, a country in which women face poor breast health outcomes due to the limited availability and unaffordability of healthcare as well as cultural stigmas related to cancer [7, 8]. The literature on developing, disseminating, and evaluating cancer education campaigns in Southeast Asia is still in a nascent stage [9]. Thus, the primary purpose of the current manuscript is to describe lessons learned from the process of disseminating a novel, culturally grounded breast cancer education intervention utilizing a pot song, an indigenous Bangladeshi art form combining visual and oral storytelling.

## Background and Need

BCa was the most common cancer and the leading cause of cancer deaths among women in Bangladesh in 2018 [10]. A nationally representative survey published in 2016 found that 81.9% of women in Bangladesh did not know about BCa and that despite existing resources, this lack of awareness about the importance of regular screening was the biggest barrier for BCa screening uptake in Bangladesh [11]. A lack of accessible cancer education resources, low socio-economic status (SES), lack of adequate healthcare facilities, and limited transportation are complex barriers adding to the lack of preventive behaviors pertaining to BCa, such as care-seeking for potential breast problems [6, 7, 12]. In addition to these general barriers, rural Bangladeshis have a specific cultural context that can inhibit breast care, such as perceptions that breast cancer is a death sentence, shame associated with talking about the breast, discomfort with male physicians, lack of support from family members, financial dependence on male relatives, and misperceptions about contagiousness [7]. As a result, care for women with breast cancer often first occurs at advanced stages of disease when available treatment is relatively ineffective or unaffordable, resulting in high rates of mortality [13]. Currently, there is no guidance in the literature on how to develop BCa education materials for rural, Asian context that are theoretically grounded and culturally situated [6]. This study aims to remedy that gap by conducting a post-test, controlled experiment in the Southern district of Khulna in Bangladesh. A non-governmental organization, *Amader Gram* (Our Village), has been established there to provide BCa care to the residents of the villages in the district. The goal of the study was to explore novel ways to encourage BCa screening among rural populations, particularly indicating the availability of a local breast care facility, *Amader Gram*, if the residents choose to get breast care in the future [14].

## Development of a Culturally Grounded Breast Cancer Intervention

The first step of our process was to conduct a formative evaluation. Key informant interviews and focus groups were conducted in Khulna, a southern division in Bangladesh. These interviews and focus groups were used to identify key cultural, linguistic, and structural factors influencing breast problem care-seeking (Table 1) [7, 14]. One key finding was that there is no specific word or words for breast cancer in the commonest native language—Bangla. Furthermore, mentioning the word for breast was often considered socially inappropriate. Access was also a key issue, with women reporting that their husbands and mothers-in-law would not support them visiting the local community clinic. Women noted that making arrangements for childcare and transportation to the clinic without family members’ support would be difficult. Thus, men and mothers-in-law were identified as being a key audience for the campaign.

**Table 1:** Sample characteristics

	Women *n* (%)	Men *n* (%)	Total *N* (%)
Number of participants	448 (45.8)	530 (54.2)	978 (100)
Marital status			
Reported	435 (97.1)	528 (99.6)	963 (98.5)
Never married	8 (1.8)	7 (1.3)	15 (1.6)
Married	414 (92.4)	514 (97.0)	928 (96.4)
Divorced	4 (0.9)	1 (0.2)	5 (0.5)
Widowed	9 (2.0)	6 (1.1)	15 (1.6)
Number of children			
Reported	433 (45.2)	524 (54.8)	957 (97.9)
0–3	375 (86.6)	450 (85.9)	825 (86.2)
4 or more	58 (13.4)	74 (14.1)	132 (13.8)

The formative research phase influenced all aspects of campaign development, including information dissemination channels, audience analysis and segmentation, and message content and design. Consistent with culturally grounded message design principles, the results of the formative research were translated into campaign elements through collaboration with community stakeholders, including community members, healthcare providers, members of non-governmental organizations, academics, and performance artists. The recommendation that emerged through this process was to disseminate BCa and breast care information in rural Bangladeshi villages via a “pot song,” an indigenous Bangladeshi art form combining visual and oral storytelling.

### Pot Song

**Fig. 1:**
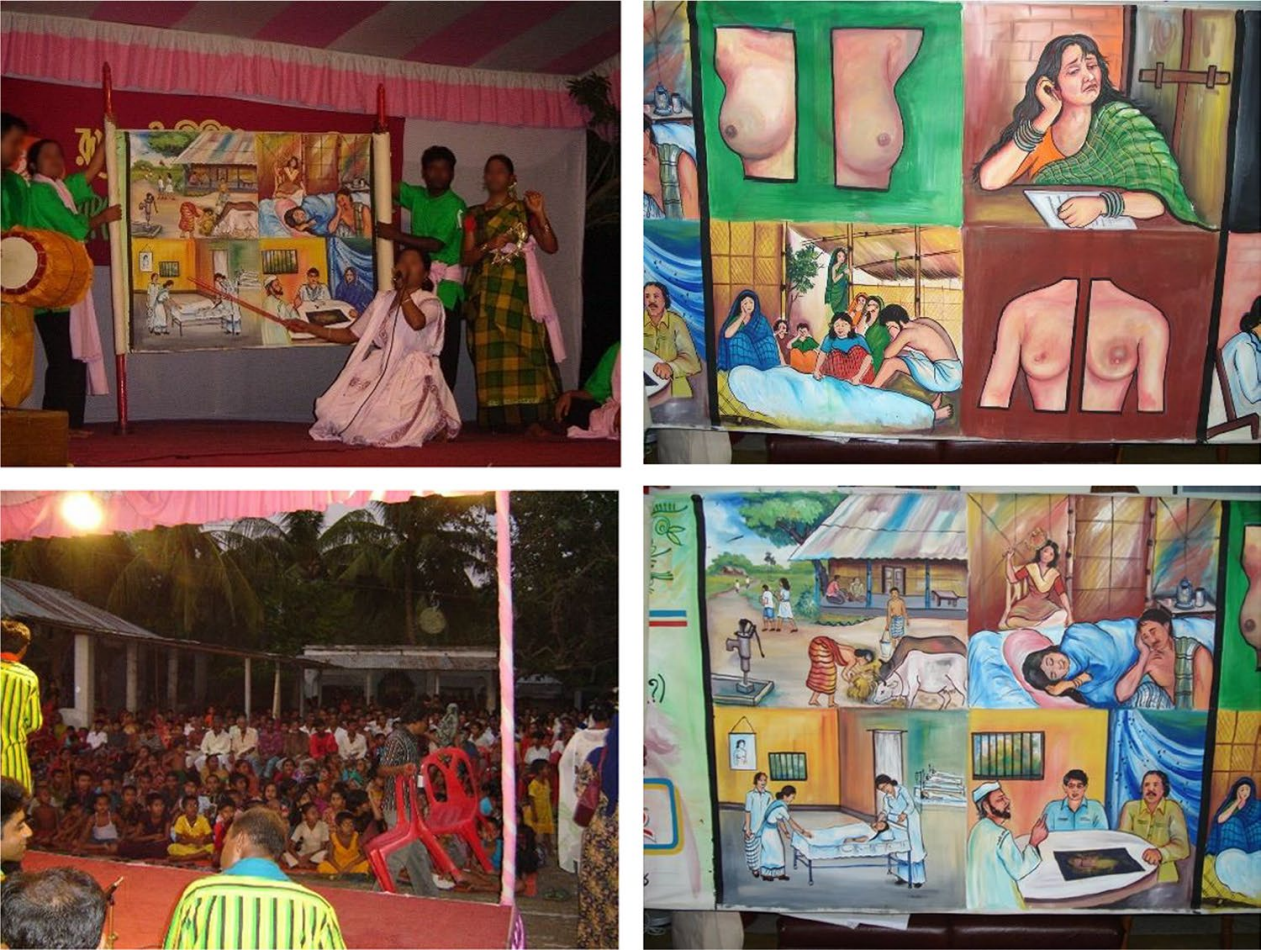
Performance of the pot song by the artists in a rural community setting

In rural Bangladesh, clay pots have been used traditionally as percussions, props, costumes (a headwear like a helmet), and characters (faces painted on the pots) in folk plays to entertain the rural residents. Pot songs are a traditional, folk-cultural performance, popular in the Southern part of Bangladesh, to promote pro-social behavioral change among people in an entertaining way. Due to their popularity, NGOs like *Rupantar* harnessed this cultural technique to create pro-social campaigns. Pot songs are a combination of folk music and a catchy, memorable lyrics, presented with choreographed dance moves and acting by rural artists. The name “pot song” is derived from the fact that the artists often use locally made, colorfully painted clay pots as part of their performance, using the pots as metaphors for a subject (e.g., breast when talking about breast cancer) or as a percussion instrument (see Fig. 1) [15].

Pot songs are created and performed by artists in community settings, with a raised platform like a theatre where the artists perform and the audience sitting in front (either on the floor or on chairs). When pot songs are performed in villages, the entire family often attends the event (i.e., men, women, in-laws, children). The artists sing as images painted on a scroll simultaneously depict the lyrics (Fig. 1). *Rupantar*, a registered non-governmental organization in Bangladesh, was hired to co-develop and disseminate a pot song about breast problems and care (see Appendix). *Rupantar* was chosen given its established history of using pot songs to communicate about a range of health and wellness topics in Bangladesh (e.g., the environment) [16]. However, there is no published literature describing the efficacy of a pot song for disseminating health communication interventions.

The co-development process included the research team sharing the results of the formative research and other feedback from stakeholders. The research team also provided information about key theoretical elements to be included in the pot song (see English translations of key message elements in Table 2). *Rupantar* provided initial translations and artistic representations of the content. Various stakeholders reviewed the drafts and collaboratively worked with *Rupantar* to identify solutions to key issues (e.g., what word to use for breast, how to represent a breast visually in a culturally appropriate way).

**Table 2:** Description of the breast cancer pot song intervention

Plot	Message aim	Selected constructs used	Example from the lyrics
“The Praise”	An introductory section to ease the audience into the topic by praising them and their knowledge about the importance of health	Severity	“One of the deadliest diseases.”
“What is Cancer”	A general description of cancer to introduce the indiscriminate nature of it	Susceptibility	“This disease attacks all — poor or rich”
“Breast Cancer”	Specifying the dangers of breast cancer, underscoring the gender-specific issues	Efficacy of screening/early treatment	“If treated at the primary stage majority of the cancer May be controlled before spreading up”
“Detection of Breast Cancer”	Detailing the benefits and processes of early detection and prompt treatment	Response efficacy of prompt treatment	“Life and money could be saved if detected and treated early”
		Knowledge	“If there is any tubercle without pain She should go to the doctor immediately”
“Relationship between Husband and Wife”	Underscoring men’s role in motivating women to be examined for breast cancer	Norms (men)	“They should be inquisitive about the physical condition of wives”
		Norms (women)	“The women are better aware and they should discuss With their husbands without being shy”
“The Urge”	Concluding with information about access for early detection and prevention	Access	“Amader Gram has taken initiative To open a health and advisory center in Khulna and Bagerhat”

### Theoretical Underpinnings

Two theories guided the design of the pot song: the extended parallel process model (EPPM) [17] and the theory of planned behavior (TPB) [18]. EPPM has been widely used in preventive message designs to increase examination of breast, skin, testicular, and cervical cancers [19–22]. EPPM predicts that increasing the perceived threat of breast cancer combined with increasing the perceived efficacy of breast care and treatment will increase intentions to seek breast care resources (Tables 3 and 4) [17]. The construct of subjective norms from TPB was also incorporated based on prior literature, showing that it is an important predictor of seeking breast care [23].

**Table 3:** Outcomes for female participants in the intervention (breast cancer) and comparison (environment) groups

Constructs (Cronbach’s alpha)	Question (Cronbach’s alpha/Spearman-Brown coefficient)	Yes *n* (%)		No *n* (%)		Chi-square tests
		Exp	Ctrl	Exp	Ctrl	
Perceived efficacy (0.70)	Perceived self-efficacy (0.60/0.61)					
	It is simple for you to go to a local health center if you have a breast problem	210 (93.8)	196 (89.9)	14 (6.3)	22 (10.1)	χ^2^ (1) = 2.18 *p* = 0.14 *n* = 442
	It is easy for you to go to a local health center if you have a breast problem	186 (83.0)	183 (89.9)	38 (17.0)	35 (16.1)	χ^2^(1) = 0.07 *p* = 0.80 *n* = 442
	Perceived response efficacy (0.64/0.64)					
	Going to the local health center is a good way to find out if you have a breast problem	205 (91)	202 (93)	19 (8.5)	14 (6.5)	χ^2^(1) = 0.63 *p* = 0.43 *n* = 440
	Going to a local health center will help you find out if you have a breast problem	204 (93.2)	180 (84.9)	15 (6.8)	32 (15.1)	χ^2^(1) = 7.54 ***p* = 0.01** Cramer’s *V* = 0.13 *n* = 431
Perceived threat (0.50)	Perceived severity (0.66/0.66)					
	Breast disease that is not detected early is a serious threat to your health	209 (94.6)	202 (93.1)	12 (5.4)	15 (6.9)	χ^2^(1) = 0.42 *p* = 0.52 *n* = 438
	Breast disease that is not detected early is bad for your health	211 (95.5)	204 (94.4)	10 (4.5)	12 (5.6)	χ^2^(1) = 0.24 *p* = 0.62 *n* = 437
	Perceived susceptibility (0.58/0.58)					
	You could get breast disease someday	202 (90.6)	186 (85.3)	21 (9.4)	32 (14.7)	χ^2^(1) = 2.89 *p* = 0.09 *n* = 441
	It is possible that you may get breast disease someday	180 (80.4)	175 (80.3)	44 (19.6)	43 (19.7)	χ^2^(1) = 0.00 *p* = 0.98 *n* = 442
	Perceived norms (− 1.08/ − 1.18)					
	Your family would support you are going to a local health center if you had a breast problem	217 (96.9)	210 (96.8)	7 (3.1)	7 (3.2)	χ^2^(1) = 0.00 *p* = 0.95 *n* = 441
	If you had a breast problem, your family would want you to go to a local health center	16 (7.2)	18 (8.4)	205 (92.8)	196 (91.6)	χ^2^(1) = 0.21 *p* = 0.65 *n* = 435
	Perceived cost					
	Going to a local health center to get treatment for breast problems has a low cost	55 (25.1)	48 (22.3)	164 (74.9)	167 (77.7)	χ^2^(1) = 0.47 *p* = 0.50 *n* = 434
	Perceived access					
	There is a local health center close by where you can get treatment for breast problems	155 (70.8)	133 (63.3)	64 (29.2)	77 (36.7)	χ^2^(1) = 2.69 *p* = 0.10 *n* = 429
	Behavioral intention					
	It is likely that you will go to a breast problem clinic in Bagerhat Town	213 (95.5)	211 (98.1)	10 (4.5)	4 (1.9)	χ^2^(1) = 2.44 *p* = 0.12 *n* = 438
	Attitude					
	The next time you have a breast problem, it is likely that you will go to a local health center that treats breast problems	179 (80.6)	173 (80.8)	43 (19.4)	41 (19.2)	χ^2^(1) = 0.00 *p* = 0.96 *n* = 436
	Knowledge					
	Do you know that there is a breast problem clinic in Bagerhat Town?	58 (26.1)	35 (16.3)	164 (73.9)	180 (83.7)	χ^2^(1) = 6.32 ***p* = 0.01** *n* = 437 Cramer’s *V* = 0.12

Note: *Exp.* experiment, *Ctrl* control; response options collapsed from four to one merging yes and mostly yes into one and no and mostly no into another

**Table 4:** Outcomes for male participants in the intervention (breast cancer) and comparison (environment) groups

Constructs (Cronbach’s alpha)	Question (Cronbach’s alpha/Spearman-Brown coefficient)	Yes *n* (%)		No *n* (%)		Chi-square tests
		Exp	Ctrl	Exp	Ctrl	
Perceived efficacy (0.56)	Perceived self-efficacy (0.33/0.34)					
	It is simple for your wife to go to a local health center if she is having a breast problem	261 (90.0)	216 (90.0)	29 (10.0)	24 (10.6)	χ^2^ (1) = 0.00 *p* = 1.00 *n* = 530
	It is easy for your wife to go to a local health center if she has a breast problem	256 (88.3)	199 (82.9)	34 (11.7)	41 (17.1)	χ^2^(1) = 3.11 *p* = 0.08 *n* = 530
	Perceived response efficacy (0.68/0.70)					
	Going to the local health center is a good way for your wife to find out if she has a breast problem	286 (99.0)	232 (96.7)	3 (1)	8 (3.3)	χ^2^(1) = 3.39 *p* = 0.07 *n* = 529
	Going to a local health center will help your wife find out if she has a breast problem	280 (96.9)	226 (94.6)	9 (3.1)	13 (5.4)	χ^2^(1) = 1.77 *p* = 0.18 *n* = 528
Perceived threat (0.67)	Perceived severity (0.73/0.73)					
	Breast disease that is not detected early is a serious threat to your wife’s health	287 (99.3)	227 (94.6)	2 (0.7)	13 (5.4)	χ^2^(1) = 10.62 ***p* = 0.00** Cramer’s *V* = 0.14 *n* = 529
	Breast disease that is not detected early is bad for your wife’s health	287 (99.3)	228 (95.4)	2 (0.7)	11 (4.6)	χ^2^(1) = 8.33 ***p* = 0.00** Cramer’s *V* = 0.13 *n* = 528
	Perceived susceptibility (0.61/0.61)					
	Your wife could get breast disease someday	252 (86.9)	210 (87.5)	38 (13.1)	30 (12.5)	χ^2^(1) = 0.04 *p* = 0.84 *n* = 530
	It is possible that your wife may get breast disease someday	263 (91.0)	210 (87.5)	26 (9.0)	30 (12.5)	χ^2^(1) = 1.70 *p* = 0.19 *n* = 529
	Perceived norms (0.83/0.84)					
	You would support your wife going to a local health center if she had a breast problem	289 (99.7)	233 (97.1)	1 (0.3)	7 (2.9)	χ^2^(1) = 5.84 ***p* = 0.02** Cramer’s *V* = 0.11 *n* = 530
	You would support your wife going to a local health center if she had a breast problem	285 (98.6)	230 (96.2)	4 (1.4)	9 (3.8)	χ^2^(1) = 3.09 *p* = 0. 08 *n* = 528
	Perceived cost					
	Going to a local health center to get treatment for breast problems has a low cost	247 (86.7)	204 (85.4)	38 (13.3)	35 (14.6)	χ^2^(1) = 0.19 *p* = 0.67 *n* = 524
	Perceived access					
	There is a local health center close by where your wife can get treatment for breast problems	215 (74.4)	179 (74.9)	74 (25.6)	60 (25.1)	χ^2^(1) = 0.02 *p* = 0.90 *n* = 528
	Behavioral intention					
	The next time your wife has a breast problem, it is likely that you will encourage her to go to a local health center that treats breast problems	76 (26.2)	57 (60.1)	214 (73.8)	182 (76.2)	χ^2^(1) = 0.39 *p* = 0.53 *n* = 529
	Attitude					
	The next time your wife has a breast problem, it is likely that you will take her to a local health center that treats breast problems	220 (75.9)	176 (73.6)	70 (24.1)	63 (26.4)	χ^2^(1) = 0.34 *p* = 0.56 *n* = 529
	Knowledge					
	Do you know that there is a breast problem clinic in Bagerhat Town?	109 (37.8)	91 (38.1)	179 (62.2)	148 (61.9)	χ^2^(1) = 0.00 *p* = 0.96 *n* = 527

## Intervention Implementation

### Overview of Intervention Design

The efficacy of the intervention for increasing breast care-seeking intentions was evaluated in a group-randomized, post-test-only experimental design. The experiment had two arms. Participants in the treatment condition viewed a pot song on the topic of breast problems. Participants in the attention control condition viewed a pot song on the topic of the environment. Exposing both groups to the pot song enabled the team to control for potential effects due to intervention delivery format (i.e., message source, delivery style). The intervention was evaluated in 18 villages in 2012 in Khulna, in the southwestern region of Bangladesh. Of the participating villages, ten were randomly assigned to the experiment condition (i.e., breast cancer pot song), and eight were randomly assigned the attention control condition (i.e., environmental pot song). The pot song used in the attention control condition had been developed and performed by *Rupantar* for a previous project to raise awareness about protecting the environment. The evaluation methods used in the current study were developed by the intervention development team (A.R., H.S., and J.K.). The instrument was administered by an expert in community engagement in region (R.S.). Given the lack of similar research in the literature, the team focused on collecting data in the most culturally valid method possible.

### Instrumentation

The study team could not identify any validated measures for assessing intervention effectiveness in Bangla. Thus, the Risk Behavior Diagnosis (RBD) scale was translated and adapted in bangla [24]. The RBD has been successfully adapted in similar situations for other non-English-speaking populations [25]. The questions and response choices were developed in English and then translated into Bangla. Each item was asked in a statement form with four options (agree a lot, agree a little, disagree a little, disagree a lot). A pilot test of the instruments was conducted to identify the best response choices. When participant suggestions for response choices were back-translated for validity, the English translations of the options were yes, mostly, not really, and no.

The content of the post-test questionnaire was constructed for women and adapted for men. Perceived self-efficacy, response efficacy, perceived susceptibility, severity, and subjective norms were measured with two items. For example, attitude toward breast care was measured using one item: “The next time you have (or your wife has) a breast problem, how likely is it that you will go to a local health center that treats breast problems?” The behavioral intention was also measured using one item. Women were asked, “How likely is it that you will go to a breast problem clinic in Bagerhat Town?” Men were asked, “The next time your wife has a breast problem, how likely is it that you will encourage her to go to a local health center that treats beast problems?” Necessary demographic information was also assessed.

### Procedures

The pot songs were performed in centralized locations within each village (i.e., open market, public school, or a community center) by experienced pot song artists (Fig. 1). Recruitment was conducted via procedures approved by the Institutional Review Board (IRB) of (Arizona State University) University. Amader Gram, a non-governmental organization and research partner, announced the upcoming performances in the villages, and participants voluntarily came to view the performances. After the pot song, trained community health workers (CHWs) from Amader Gram randomly selected audience members to complete a post-test questionnaire. The CHWs obtained verbal informed consent and administered the post-test questionnaire with audience members. The CHWs recorded the answers on paper copies of the questionnaire. No identifiable information was collected.

## Evaluation of the Intervention

### Process Evaluation

The process evaluation of the intervention included members of the study team observing the implementation of the intervention and the post-test questionnaire. An initial concern was that community members would not participate in the study due to cultural sensitivity regarding the visual or lyrical depictions of the breast. The observations of the study team were that the intervention and attention control pot songs were equally well attended. Furthermore, participants in both groups (*N* = 516 in the intervention group; *N* = 462 in the control group) were equally willing to participate in the post-test questionnaire. The number of participants refers to those who consented to and completed the post-test questionnaire. Attendance at the live performances were higher; however, the exact number of attendees is not available due to the fact that culturally, pot songs are performed in large, public areas without formal strategies for monitoring attendance. A second important piece of process evaluation data is how participants responded to the questionnaire administration. Every effort was taken to ask participants questions privately. However, given the social setting of the pot song, members of the study team observed that other individuals would attempt to participate in the individual interviews by listening to the questions and calling out responses. The CHWs were trained to record the verbal responses of the individual participant, but it is unknown if the presence of others influenced responses.

### Outcome Evaluation

The outcome evaluation of the intervention focused on the analysis of the quantitative post-test measures. Initial examination of the data showed non-normal distributions for the variables. As such, chi-square analyses were used to compare female (Table 3) and male (Table 4) participants in the intervention and control conditions. Key findings for the female data include that participants in the intervention group showed significantly higher perceptions of knowledge about the breast clinic than the women in the control group. One item indicated that the intervention resulted in higher response efficacy of breast care (see Table 3 for full results). Men in the intervention group showed a significantly increased perceived threat of breast cancer than the men in the control group. Men in the intervention group also reported higher perceived norms (see Table 4 for full results).

## Discussion

This study aimed to design, implement, and evaluate an intervention to promote BCa awareness and breast care in a rural Bangladeshi setting. The process evaluation indicated that the intervention successfully navigated the cultural sensitivities associated with communication about female anatomy in Bangladeshi culture. The outcome evaluation showed significantly increased knowledge among the female participants in the intervention group about local breast care services availability. Furthermore, men in the intervention group demonstrated more willingness to encourage their wives to be screened for breast problems.

Although the formative research stage resulted in the successful development of a culturally sensitive intervention, the intervention was not as efficacious in changing the antecedents of health behavior change as the team anticipated. Each component of the pot song was carefully crafted to reflect specific behavioral variables, many of which were not significantly different from participants in the control condition. Given that pot songs are regularly used by non-governmental organizations in Bangladesh for health campaigns, this study demonstrates the importance of identifying reliable ways to evaluate the efficacy of arts-based interventions in various cultural contexts, such as the pot song in Bangladesh. At present, it is unknown whether pot song interventions are not being evaluated or whether interventions without significant findings are not being published in English or Bangla.

Another important implication of this study is the importance of measurement in different languages. This study pioneered an adaptation of the RBD scale to Bangla. However, the reliability scores for the constructs were low. In the adaptation process, every attempt was made to ensure the wording of the items was similar for constructs. However, there may be cultural factors that resulted in items intended to be similar being interpreted quite differently. An example of this is perceived norms. Most women responded that they thought their husband would support them going to a clinic if they had a breast problem, yet they responded negatively to the question asking whether their husband would *want* them to go. This might indicate that women thought their husbands would support them going to the breast clinic out of obligation but would do so reluctantly. Further research is needed to develop and refine measurement instruments to measure cognitive predictors of cancer education and behavioral change in this cultural context. Such processes could be valuable for informing cancer education and detection interventions among diverse cultural groups across the globe [23].

Understanding cultural norms in different areas is an essential component of developing and improving measurement instruments. One possible reason that the intervention group did not differ from the control group on many of the outcome variables is due to acquiescence bias. Acquiescence bias refers to participants agreeing with a question regardless of the content, even when the questions are neutral [26]. The tendency to agree is more prevalent among populations with lower SES and/or that place greater importance on social desirability, collectivism, and familism [26, 27]. Given that the data are positively skewed, this could indicate a tendency for acquiescence bias. However, there is little published literature examining this phenomenon in Bangladesh or other Southeast Asian counties.

Finally, the current study focused on developing a culturally grounded intervention. However, like the adaptation of measurement instruments, data collection methods also need to be culturally grounded. Field observations showed a tension between maintaining fidelity to a data collection process focused on individual responses to a post-test questionnaire and the cultural norms of the population that expected interviews to be completed in groups. While experimental research designs typically conceptualize questionnaire completion as an individual activity, we observed that participants engaged the research staff in groups. A key area for future research is to further explore innovative strategies for administering evaluations in a manner that supports the internal validity of the research design while ensuring that the methods maintain ecological validity by remaining consistent with cultural norms. For example, it would not have been culturally appropriate in some villages for female participants to be interviewed privately by a male researcher (and vice versa). Similar interventions may want to consider mixed-methods designs to explore whether individual and group-based data collection yields similar outcomes. Other researchers interested in cancer education in remote areas should also note the logistical difficulties of collecting private responses if the intervention is deployed in a social setting and cultural norms are geared toward group rather than individual responses. Our experiences indicate that interventions administered in community locations should utilize designs that emphasize external validity for this cultural context, such as small group interviews.

## Conclusion

This manuscript describes the development, implementation, and evaluation of a culturally grounded BCa awareness and breast care education intervention for rural Bangladeshis. The intervention was carefully designed to be theoretically grounded and culturally sensitive. The process evaluation data indicated the intervention successfully provided education on a taboo topic in a culturally appropriate manner, and the outcome evaluation showed improvement in knowledge and social norms for breast care. This study also demonstrates key challenges associated with developing study procedures and measurement instruments that reflect diverse cultural contexts and languages. This study provides a framework for building a literature around culturally grounded cancer education in Southeast Asian contexts.

## Data Availability

The datasets generated during the current study are available from the corresponding author on reasonable request.

## Appendix

**Pot Song on Breast Cancer**

Composed by: Md. Elias Fakir

Concept: Reza Salim

Planning: Rafiqul Islam Khokan

Pot Painting: Sreebash Mondal

Direction: Swapan Guha

**The world is full of deadly disease; we now know its identity**

**Let us be conscious well in advance!!**

**Plot – I: The Praise**

Listen oh the learned and wise audience

Think deeply and you know that Health is above all

We must try to keep us healthy

Healthy people bring national prosperity

We will have to think of why we are attacked by disease

And how to get cured

Consciousness is a pre-condition for keeping well

And remain healthy

Cancer is one of the deadliest diseases

Now we want to narrate on the horrific disease- The Cancer!!

**Plot – 2: What is Cancer?**

Cancer is one of the deathly diseases

It is not possible for us to understand

When and how we will be attacked

This disease attacks all — poor or rich

Human body is made of billions of cells

Like bricks of a building

Abnormal cell development in the body

Leads to cancer

More than four million people die due to cancer

Every year in the world

And there are two types of tumors-Benign and Malignant

The Malignant one is the cancer

It brings the unwanted death and has no remedies (if it spreads too much)!!

**Plot – 3: Breast Cancer**

Any body may be attacked by cancer irrespective of race or sex

But the females are attacked more than male (for certain cancers)

Usually suffering from uterine and breast cancer

Due to shyness, the women do not usually disclose

Their problem till seriousness arise

When even doctors fail to provide remedy and

Death becomes inevitable

The enumeration shows

Death from (breast and uterine) cancer is more in female than the male

If treated at the primary stage, majority of the cancer

May be controlled before spreading up

Once it is spread, death approaches quietly!!

**Plot – 4: Detection of breast cancer**

In Bangladesh, one out of five women suffer from breast cancer

Life and money could be saved if detected and treated early

To do so, the women should not feel shy

Rather check for the tumor by herself

Once they have crossed the age of thirty-five

If there is any tubercle without pain

She should go to the doctor immediately

There are many healthcare centers —

Serving people free of cost

Even at the Upazilla level

None should be shaky for any disease

Shyness will be ruining!!

**Plot – 5: Relationship between husband and wife**

Our society is such that discusses any happening seriously

There are discussions worldwide on certain diseases

But we are not thinking of this breast cancer

The leaders of our society are thinking on women’s issues

We hope they will think on the breast cancer issue

The husbands should be more careful on their wives

They should be inquisitive about the physical condition of wives

The women are better aware, and they should discuss

With their husbands without being shy

If there is any doubt, they should quickly consult a lady doctor

And take advice of the doctor to save life

**Plot – 6: The Urge**

Man is the best of all living creatures of the world

Man becomes cautious on any problem

In cancer, it is possible to live being careful

If the women feel pain in their sex organs

Being shy, they do not express it

In the end, it becomes the cause of death

Under this situation

Amader Gram has taken initiative

To open a health and advisory center in Khulna and Bagerhat

The doctor of the center will give advice

If you are doubtful about breast cancer

Please come to the health center

Where you will be served cordially

You may also visit the government hospital nearby

To get yourself checked

Do not become shy and conceal your diseases

That may have fatal consequences, including death!!

**Female Interview Guide**

1. It is simple for you to go to a local health center if you have a breast problem.
  - Agree a lot
  - Agree a little
  - Disagree a little
  - isagree a lot
2. You could get breast disease someday.
  - Agree a lot
  - Agree a little
  - Disagree a little
  - Disagree a lot
3. Breast disease that is not detected early is a serious threat to your health.
  - Agree a lot
  - Agree a little
  - Disagree a little
  - Disagree a lot
4. Going to the local health center is a good way to find out if you have a breast problem.
  - Agree a lot
  - Agree a little
  - Disagree a little
  - Disagree a lot
5. Your family would support you are going to a local health center if you had a breast problem.
  - Agree a lot
  - Agree a little
  - Disagree a little
  - Disagree a lot
6. It is easy for you to go to a local health center if you have a breast problem.
  - Agree a lot
  - Agree a little
  - Disagree a little
  - Disagree a lot
7. It is possible that you may get breast disease someday.
  - Agree a lot
  - Agree a little
  - Disagree a little
  - Disagree a lot
8. Breast disease that is not detected early is bad for your health.
  - Agree a lot
  - Agree a little
  - Disagree a little
  - Disagree a lot
9. Going to a local health center will help you find out if you have a breast problem.
  - Agree a lot
  - Agree a little
  - Disagree a little
  - Disagree a lot
10. If you had a breast problem, your family would want you to go to a local health center.
  - Agree a lot
  - Agree a little
  - Disagree a little
  - Disagree a lot
11. Going to a local health center to get treatment for breast problems has a low cost.
  - Agree a lot
  - Agree a little
  - Disagree a little
  - Disagree a lot
12. There is a local health center close by where you can get treatment for breast problems.
  - Agree a lot
  - Agree a little
  - Disagree a little
  - Disagree a lot
13. Do you have any breast problems right now (that is, lumps or changes in color or size)?
  - Yes
  - No (skip to question 14)
14. If yes to #13: How likely is it that you will go to a breast problem clinic in Bagerhat Town?
  - Definitely will go
  - Probably will go
  - Probably will not go
  - Definitely will not go
15. The next time you have a breast problem, how likely is it that you will go to a local health center that treats breast problems?
  - Definitely will go
  - Probably will go
  - Probably will not go
  - Definitely will not go
16. How old are you?
  - _______ years old
17. Are you single (never married), married, divorced, or widowed?
  - Single, never married (skip to question 18)
  - Married
  - Divorced
  - Widowed
18. If married, divorced, or widowed, do you have any children (and if so, how many)?
  - 0
  - 1
  - 2
  - 3
  - 4
  - 5
  - 6 or more
19. In the last year, how many times did you go to a clinic or to see a doctor or Apa?
  - 0 times in the last year
  - 1 or 2 times in the last year
  - 3 or 4 times in the last year
  - 5 or more times in the last year
20. Do you know that there is a breast problem clinic in Bagerhat Town?
  - Yes
  - No
21. Please list the symptoms of breast cancer. [Note to interviewer: Please follow up with “anything else” until they are done listing new symptoms.]
  - Change in size/swelling
  - Change in color
  - Change in shape
  - Lump
  - Illness or death
  - Did not know/could not list any symptoms
  - Listed only wrong symptoms

**Interviewer Notes/Comments:**

Location/village: ______________________________

Date/time completed: ______________________________

Interviewer name: ______________________________

**Male Interview Guide**

1. It is simple for your wife to go to a local health center if she is having a breast problem.
  - Agree a lot
  - Agree a little
  - Disagree a little
  - Disagree a lot
2. Your wife could get breast disease someday.
  - Agree a lot
  - Agree a little
  - Disagree a little
  - Disagree a lot
3. Breast disease that is not detected early is a serious threat to your wife's health.
  - Agree a lot
  - Agree a little
  - Disagree a little
  - Disagree a lot
4. Going to the local health center is a good way for your wife to find out if she has a breast problem.
  - Agree a lot
  - Agree a little
  - Disagree a little
  - Disagree a lot
5. You would support your wife going to a local health center if she had a breast problem.
  - Agree a lot
  - Agree a little
  - Disagree a little
  - Disagree a lot
6. It is easy for your wife to go to a local health center if she has a breast problem.
  - Agree a lot
  - Agree a little
  - Disagree a little
  - Disagree a lot
7. It is possible that your wife may get breast disease someday.
  - Agree a lot
  - Agree a little
  - Disagree a little
  - Disagree a lot
8. Breast disease that is not detected early is bad for your wife’s health.
  - Agree a lot
  - Agree a little
  - Disagree a little
  - Disagree a lot
9. Going to a local health center will help your wife find out if she has a breast problem.
  - Agree a lot
  - Agree a little
  - Disagree a little
  - Disagree a lot
10. If your wife had a breast problem, you would want her to go to a local health center.
  - Agree a lot
  - Agree a little
  - Disagree a little
  - Disagree a lot
11. Going to a local health center to get treatment for breast problems has a low cost.
  - Agree a lot
  - Agree a little
  - Disagree a little
  - Disagree a lot
12. There is a local health center close by where your wife can get treatment for breast problems.
  - Agree a lot
  - Agree a little
  - Disagree a little
  - Disagree a lot
13. The next time your wife has a breast problem, how likely is it that you will encourage her to go to a local health center that treats breast problems?
  - Definitely will encourage
  - Probably will encourage
  - Probably will not encourage
  - Definitely will not encourage
14. How old are you?
  - _______ years old
  - Single, never married (skip to question 17)
  - Married
  - Divorced
  - Widowed
15. If married, divorced, or widowed, do you have any children (and if so, how many)?
  - 0
  - 1
  - 2
  - 3
  - 4
  - 5
  - 6 or more
16. Do you know that there is a breast problem clinic in Bagerhat Town?
  - Yes
  - No
17. Please list the symptoms of breast cancer. [Note to interviewer: Please follow up with “anything else” until they are done listing new symptoms.]
  - Change in size/swelling
  - Change in color
  - Change in shape
  - Lump
  - Illness or death
  - Did not know/could not list any symptoms
  - Listed only wrong symptoms

**Interviewer Notes/Comments:**

Location/village: ______________________________

Date/time completed: ______________________________

Interviewer name: ______________________________

## Code Availability

Codes used to analyze the data generated during the study are available from the first author on reasonable request.
